# Myomatous erythrocytosis syndrome associated with an 8-kg uterine leiomyoma: a case report of diagnostic evaluation and perioperative management

**DOI:** 10.1016/j.crwh.2026.e00853

**Published:** 2026-09-10

**Authors:** Naoyuki Ida, Shoji Nagao, Ichiro Ueki, Yoshinori Tani, Hisashi Masuyama

**Affiliations:** Department of Obstetrics and Gynecology, Okayama University Graduate School of Medicine, Dentistry and Pharmaceutical Sciences, 2-5-1 Shikata-cho, Kita-ku, Okayama 700-8558, Japan

**Keywords:** Myomatous erythrocytosis syndrome, Giant uterine leiomyoma, Secondary erythrocytosis, Erythropoietin, Therapeutic phlebotomy, Perioperative management

## Abstract

Myomatous erythrocytosis syndrome (MES) is a rare condition in which erythrocytosis associated with uterine leiomyoma resolves after tumor removal. A 55-year-old woman with a giant uterine mass that had continued to enlarge despite long-term leuprorelin treatment was referred for marked erythrocytosis. Treatment-related amenorrhea made the timing of natural menopause uncertain. Magnetic resonance imaging showed a heterogeneous uterine mass exceeding 30 cm. Hemoglobin was 20.1 g/dL and serum erythropoietin (EPO) was 45.4 mIU/mL. BCR-ABL1 testing was negative, and no JAK2 V617F, JAK2 exon 12, CALR, or MPL mutation was detected, supporting secondary rather than clonal erythrocytosis. Ten weekly therapeutic phlebotomies reduced hemoglobin to 15.8 g/dL. Total abdominal hysterectomy with bilateral salpingo-oophorectomy was performed after multidisciplinary perioperative planning. Prominent omental collateral vessels and markedly dilated ovarian veins increased surgical complexity. Because the giant tumor restricted posterior pelvic access, hysterectomy was completed using a retrograde approach. The 8060-g specimen was histologically confirmed as a benign uterine leiomyoma, and superficial cystic structures were identified as D2–40-positive dilated lymphatic channels. No thrombotic event occurred. At one-month follow-up, hemoglobin and serum EPO had decreased to 11.4 g/dL and 16.0 mIU/mL, respectively, without recurrent erythrocytosis. Although tumor EPO expression was not evaluated, postoperative normalization of serum EPO and resolution of erythrocytosis supported the clinical diagnosis of MES. MES should be considered in patients with uterine leiomyoma and unexplained erythrocytosis; hematologic evaluation, preoperative phlebotomy, and multidisciplinary surgical planning may enable safe treatment.

## Highlights

• An 8060-g uterine leiomyoma was associated with myomatous erythrocytosis.
• Molecular testing supported secondary rather than clonal erythrocytosis.
• Ten staged phlebotomies reduced hemoglobin from 20.1 to 15.8 g/dL.
• Multidisciplinary planning enabled safe removal despite distorted anatomy.
• Hemoglobin and serum erythropoietin levels normalized after hysterectomy.

## 1 Introduction

Myomatous erythrocytosis syndrome (MES) is a rare condition defined by erythrocytosis in the presence of a uterine leiomyoma and normalization of hematologic values after tumor removal [1], [2], [3], [4], [5], [6], [7]. Increased erythropoietin (EPO) production by leiomyoma tissue has been demonstrated in some patients and proposed as a possible explanation for MES [1], [8]. However, an elevated circulating EPO level alone does not establish tumor production, and the underlying mechanism cannot be determined without tumor-specific evidence.

Clinically, marked erythrocytosis requires systematic evaluation for polycythemia vera, other myeloproliferative neoplasms, and secondary causes before it can be attributed to a uterine leiomyoma [9], [10], [11]. Management also requires attention to the risks associated with hyperviscosity and thromboembolism. When the tumor is extremely large, extensive collateral vessels, venous engorgement, and distortion of normal pelvic anatomy may create additional surgical challenges.

This report presents a case of MES associated with an 8060-g uterine leiomyoma. It focuses on the diagnostic evaluation of erythrocytosis, staged preoperative phlebotomy, and operative strategies that enabled safe removal of an extremely large pelvic tumor.

## 2 Case Presentation

A 55-year-old woman (gravida 1, para 1) with a history of one cesarean delivery was referred for evaluation of a giant uterine mass and marked erythrocytosis. Approximately 15 years before presentation, she had undergone uterine artery embolization for uterine leiomyomas, which initially resulted in tumor shrinkage. Approximately 10 years before presentation, two cervical leiomyomas measured 4.3 and 4.7 cm. However, around seven years before presentation, the uterus had enlarged to approximately 19 × 15 cm and reached the level of the umbilicus.

She subsequently remained under follow-up at the treating institution and, according to the available referral information and the patient's account, received leuprorelin for approximately seven years until transfer of care. The precise dose, administration interval, and any interruptions in treatment could not be verified because the treating institution had subsequently closed. Despite medical treatment, the tumor progressively enlarged. The patient recalled that her last spontaneous menstrual period occurred at 47 years of age; however, the timing of natural menopause could not be determined because of prolonged gonadotropin-releasing hormone agonist-induced amenorrhea.

**Fig. 1:**
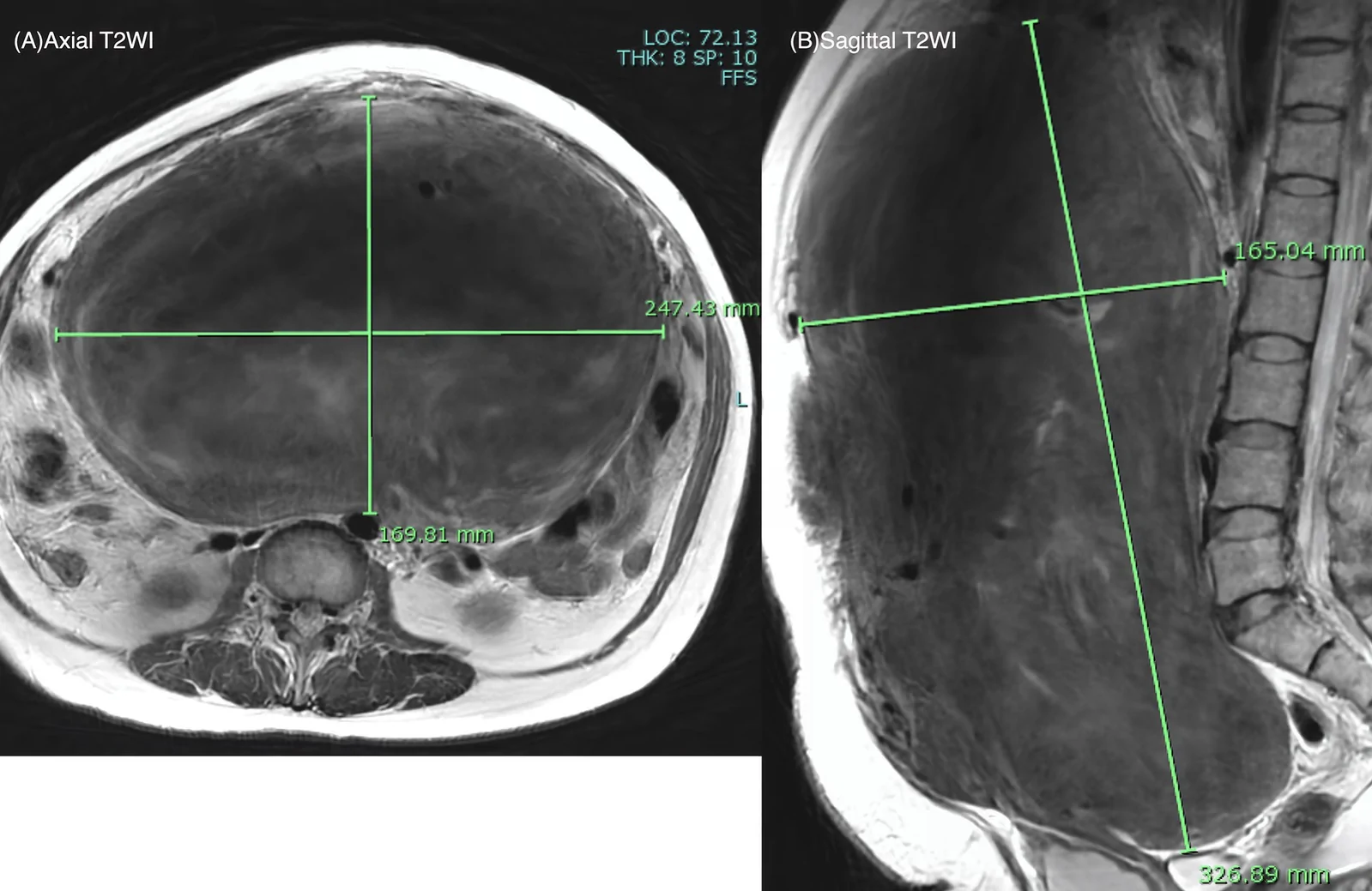
Preoperative magnetic resonance imaging findings. T2-weighted images showing a giant uterine mass with heterogeneous signal intensity, measuring approximately 32.7 × 24.7 × 17.0 cm. (A) Axial view. (B) Sagittal view.

Approximately five months before surgery, closure of the treating institution prompted referral to another hospital. Magnetic resonance imaging performed at the initial visit demonstrated a heterogeneous uterine mass measuring approximately 32.7 × 24.7 × 17.0 cm (Fig. 1). Surgery was initially planned at that hospital; however, preoperative laboratory testing revealed a hemoglobin concentration of 20.1 g/dL. She was therefore referred to an institution where hematological evaluation and perioperative management were available.

Serum erythropoietin (EPO) was elevated at 45.4 mIU/mL (reference range, 4.2–23.7 mIU/mL). BCR-ABL1 testing was negative, and no JAK2 V617F, JAK2 exon 12, CALR, or MPL mutation was detected. Together with the elevated serum EPO level, these findings made polycythemia vera and other common clonal myeloid neoplasms unlikely and supported a diagnosis of secondary erythrocytosis.

Because the tumor had continued to enlarge despite long-term leuprorelin treatment, uterine sarcoma remained a concern. Computed tomography and fluorodeoxyglucose positron emission tomography/computed tomography performed approximately three months before surgery showed no findings clearly suggestive of malignancy. Only mild fluorodeoxyglucose uptake was observed within the mass, with a maximum standardized uptake value of 2.8. Nevertheless, malignancy could not be completely excluded preoperatively.

To reduce hyperviscosity and perioperative thrombotic risk, therapeutic phlebotomy was performed weekly for 10 weeks. Six 200-mL sessions were followed by four 300-mL sessions, reducing the hemoglobin concentration from 20.1 to 15.8 g/dL before surgery.

**Fig. 2:**
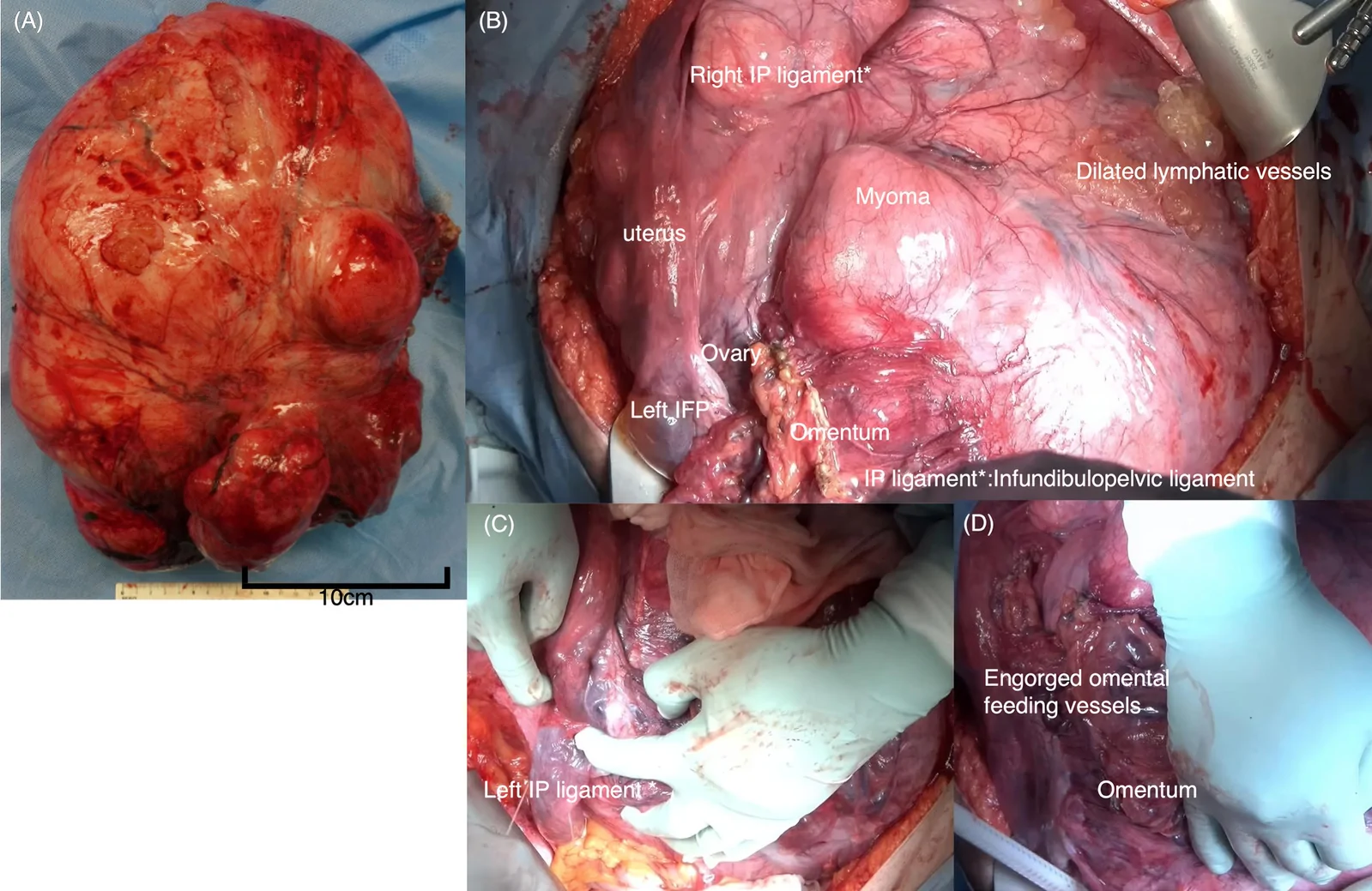
Intraoperative findings and gross specimen. (A) Gross appearance of the resected uterus containing a giant leiomyoma weighing 8060 g (scale bar, 10 cm). (B) Intraoperative view of the giant leiomyoma showing dilated superficial lymphatic vessels on the tumor surface and its relationship to the uterus, left ovary, bilateral infundibulopelvic ligaments, and omentum. (C) Markedly dilated vessels within the left infundibulopelvic ligament. (D) Engorged omental feeding vessels supplying the tumor.

Following hematological optimization, total abdominal hysterectomy with bilateral salpingo-oophorectomy was performed after placement of bilateral ureteral stents. A massive uterine tumor occupied most of the abdominal cavity. Prominent collateral feeding vessels arose from the omentum, and both ovarian veins were markedly dilated, to approximately 4 cm. Multiple dilated superficial vessels were also observed on the tumor surface (Fig. 2). Vascular control was achieved by sequential ligation of the omental feeding vessels, infundibulopelvic ligaments, and cardinal ligaments. The ureters were mobilized laterally. Because the giant tumor restricted access to the posterior pelvis, a retrograde approach was used to complete the hysterectomy. The operative time was 3 h 3 min, and the estimated blood loss was 1080 mL. The resected specimen weighed 8060 g.

**Fig. 3:**
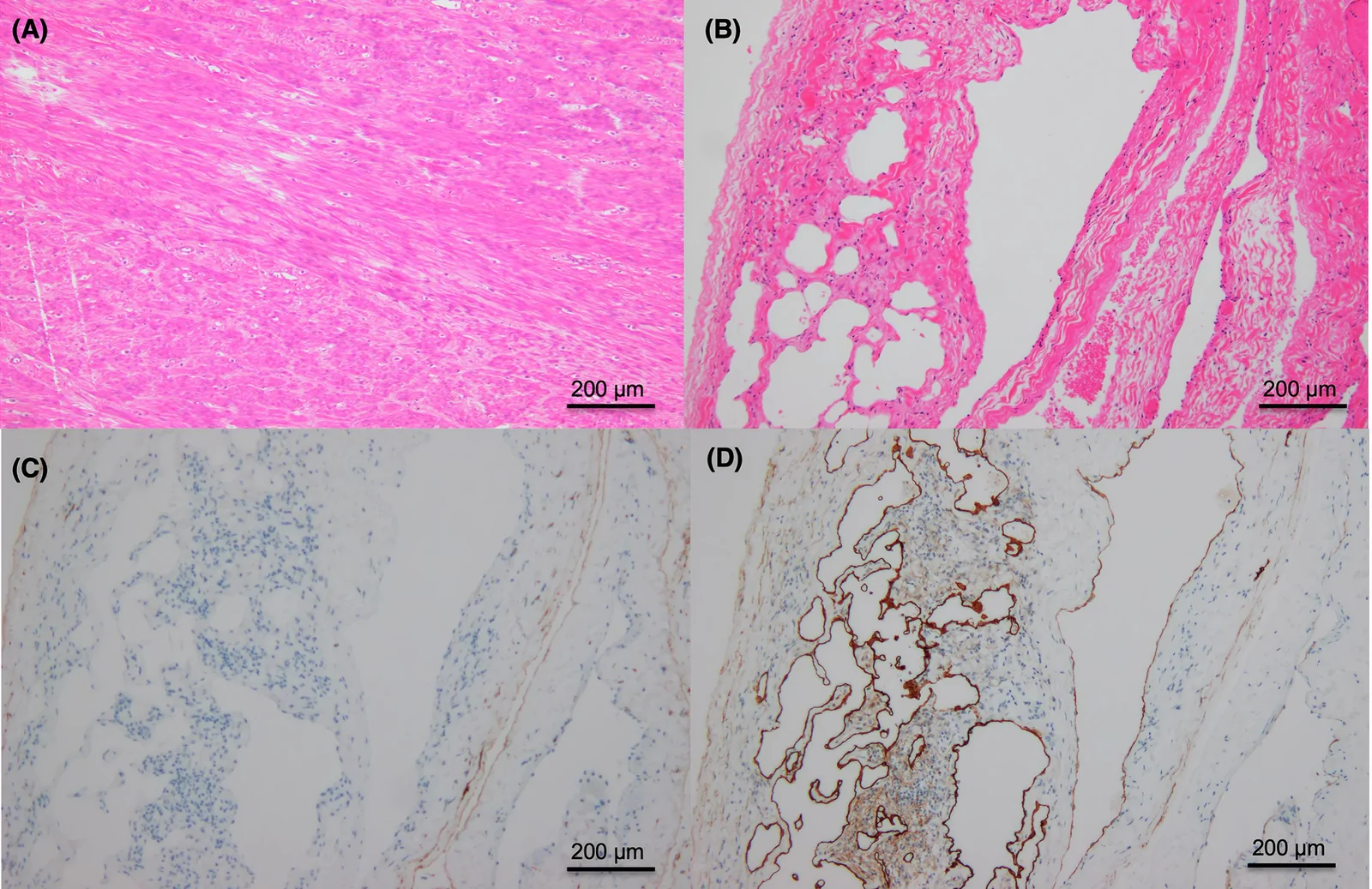
Histopathological findings of the uterine leiomyoma and superficial cystic structures. (A) Hematoxylin and eosin staining of the uterine tumor showed intersecting fascicles of bland spindle cells with minimal cytological atypia and rare mitotic figures, consistent with leiomyoma. (B) Hematoxylin and eosin staining of the superficial cystic structures showed multiple dilated channels lined by flattened endothelial cells. Immunohistochemically, the lining cells were negative for cytokeratin AE1/AE3 (C) and positive for D2–40 (D), confirming that the structures represented dilated lymphatic channels. Scale bars, 200 μm.

Histopathological examination confirmed a benign uterine leiomyoma. The superficial cystic structures were positive for D2–40 and negative for cytokeratin AE1/AE3, confirming that they represented dilated lymphatic channels (Fig. 3). EPO expression was not evaluated in the tumor tissue.

**Fig. 4:**
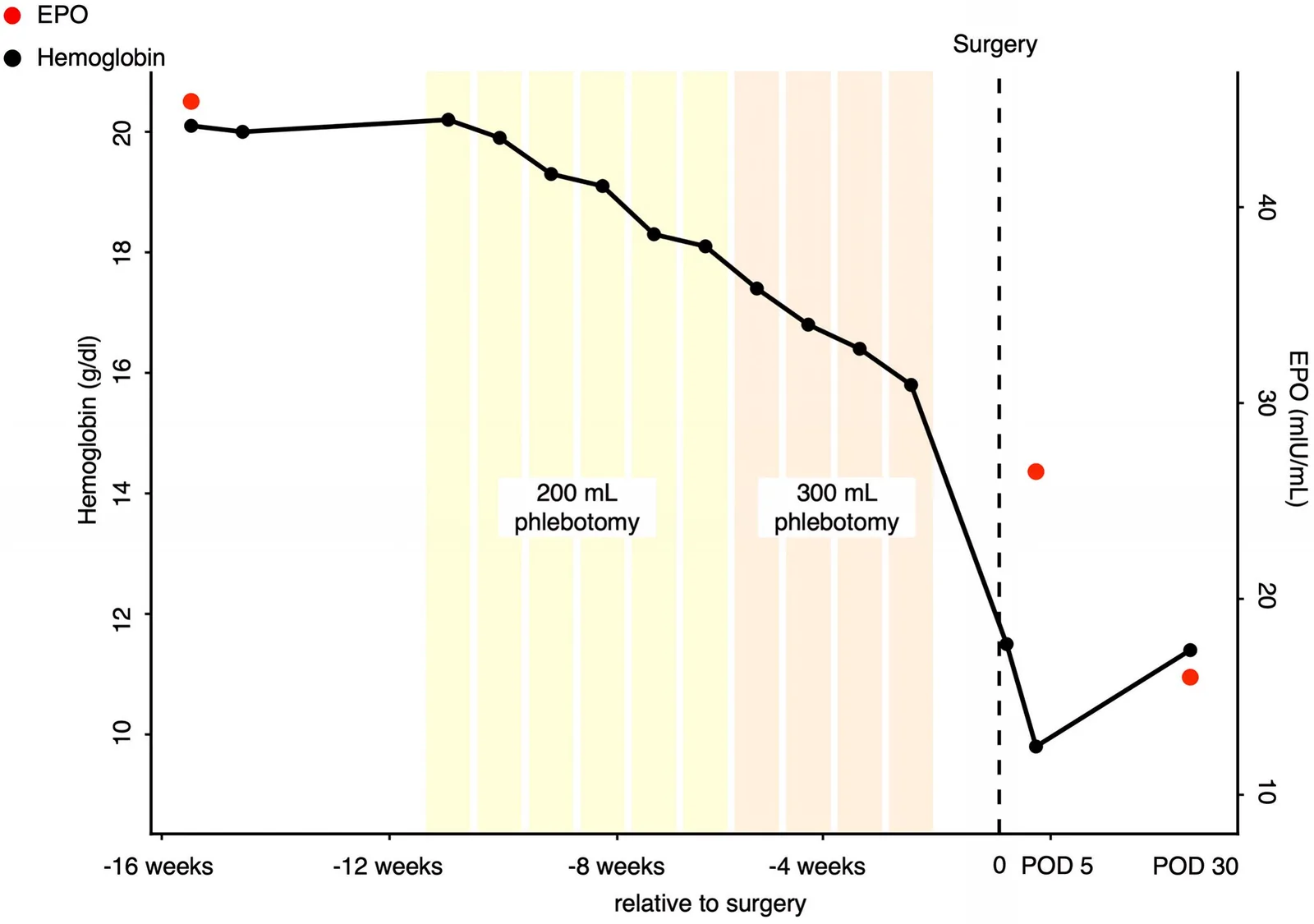
Temporal changes in hemoglobin and serum erythropoietin levels during preoperative phlebotomy and after tumor removal. Black circles connected by a solid line represent hemoglobin concentrations, and red circles represent serum erythropoietin (EPO) levels. The pale-yellow and pale-orange bands indicate six 200-mL and four 300-mL therapeutic phlebotomy sessions, respectively. The vertical dashed line denotes the day of surgery (day 0), and the horizontal axis shows time relative to surgery (POD, postoperative day). Serum EPO had returned to the reference range by postoperative day 30. EPO, erythropoietin. (For interpretation of the references to colour in this figure legend, the reader is referred to the web version of this article.)

The hemoglobin concentration decreased to 11.3 g/dL on postoperative day 1 and 9.8 g/dL on postoperative day 5. Serum EPO also decreased postoperatively to 26.5 mIU/mL. Enoxaparin was administered from postoperative day 1 until discharge on postoperative day 6, and no thrombotic event occurred. At one-month follow-up, the patient remained asymptomatic; her hemoglobin concentration was 11.4 g/dL, and serum EPO had normalized to 16.0 mIU/mL (Fig. 4), with no evidence of recurrent erythrocytosis. No further gynecological follow-up was considered necessary after confirmation of the benign diagnosis. A further hematology follow-up visit, including repeat measurement of hemoglobin and serum EPO, was scheduled for 3 months later.

## 3 Discussion

Myomatous erythrocytosis syndrome (MES) is clinically defined by the coexistence of erythrocytosis and uterine leiomyoma, followed by resolution of the erythrocytosis after tumor removal [1], [2], [3], [4], [5], [6], [7]. In the present case, marked erythrocytosis was associated with an 8060-g uterine leiomyoma, and both hemoglobin and serum EPO decreased after hysterectomy. These findings supported the clinical diagnosis of MES. At 8060 g, the tumor burden exceeded the reported mean leiomyoma weight of approximately 4.9 kg in patients with MES [4] and represents an unusually large MES-associated leiomyoma.

An important aspect of this case was the hematologic differential diagnosis. Evaluation of erythrocytosis requires distinction between polycythemia vera and secondary erythrocytosis [9], [11]. Polycythemia vera is strongly associated with JAK2 V617F or JAK2 exon 12 mutations and is usually accompanied by a subnormal serum EPO level [9], [10]. In this patient, the absence of these JAK2 mutations and the elevated serum EPO level made polycythemia vera unlikely. Negative BCR-ABL1, CALR, and MPL testing further reduced the likelihood of another clonal myeloid neoplasm. Other causes of secondary erythrocytosis, including chronic hypoxemia, sleep apnea, smoking or carbon monoxide exposure, cardiopulmonary disease, renal disease, medication-related erythrocytosis, and other EPO-associated tumors, should also be considered during the diagnostic evaluation [9], [11]. An elevated serum EPO level alone cannot identify its source. In the present case, the resolution of erythrocytosis and return of serum EPO to the reference range at one-month follow-up provided the strongest clinical evidence linking the erythrocytosis to the uterine leiomyoma.

The extreme tumor burden created additional perioperative challenges. Prominent omental collateral vessels, ovarian veins dilated to approximately 4 cm, and multiple superficial lymphatic channels were encountered intraoperatively. D2–40 positivity and cytokeratin AE1/AE3 negativity confirmed that the superficial cystic structures represented dilated lymphatic channels. These findings do not establish a specific mechanism for MES but were clinically important because they indicated markedly altered anatomy and a substantial risk of bleeding. Preoperative ureteral stent placement, early control of the omental and adnexal vessels, lateral mobilization of the ureters, and use of a retrograde approach allowed the tumor to be removed despite restricted access to the posterior pelvis.

Therapeutic phlebotomy is not routinely indicated for all forms of secondary erythrocytosis, and management should primarily address the underlying cause [11]. In this case, however, the hemoglobin level was 20.1 g/dL, and extensive surgery for a highly vascular giant tumor was planned. Staged phlebotomy was therefore used as an individualized bridging strategy to reduce hyperviscosity and perioperative thrombotic risk. Ten weekly sessions reduced hemoglobin to 15.8 g/dL before surgery. Postoperative thromboprophylaxis with enoxaparin was also administered, and no thrombotic event occurred. Although this favorable outcome supports multidisciplinary perioperative planning, a single case cannot establish the optimal hemoglobin target or phlebotomy regimen for MES.

EPO production by leiomyoma tissue has been demonstrated in some previously reported cases [1], [8]. In the present case, the elevated preoperative serum EPO level and its postoperative decline were compatible with an EPO-associated process. However, EPO immunohistochemistry, measurement of EPO within the tumor, and comparison of uterine arterial and venous EPO concentrations were not performed. Therefore, direct EPO production by the leiomyoma was not demonstrated. Furthermore, the observed collateral vessels, venous dilatation, and lymphatic channels should not be interpreted as evidence of intratumoral hypoxia or as proof of a specific EPO-producing mechanism. The short follow-up period and the inherent inability of a single case to establish causality are additional limitations.

This case demonstrates that MES should be considered when marked erythrocytosis occurs in a woman with a large uterine mass. Detailed hematologic evaluation is necessary before attributing erythrocytosis to a leiomyoma. In patients with extreme tumor burden, individualized hematologic optimization and careful multidisciplinary surgical planning may facilitate safe definitive treatment.

## 4 Conclusion

This case describes clinically diagnosed MES associated with an unusually large uterine leiomyoma. Staged preoperative phlebotomy and surgical planning enabled safe tumor removal despite extensive collateral vessels and distorted pelvic anatomy.

## Contributors

Naoyuki Ida contributed to patient care, conception of the case report, acquiring and interpreting of the data, drafting the manuscript, undertaking the literature review, and revising the article critically for important intellectual content.

Shoji Nagao contributed to conception of the case report, interpreting the data, and revising the article critically for important intellectual content.

Ichiro Ueki contributed to patient care, acquiring and interpreting the data, and revising the article critically for important intellectual content.

Yoshinori Tani contributed to patient care, acquiring and interpreting the data and revising the article critically for important intellectual content.

Hisashi Masuyama contributed to conception of the case report, interpreting the data and revising the article critically for important intellectual content.

All authors approved the final submitted manuscript.

## Patient consent

Written informed consent was obtained from the patient for publication of the case report and accompanying images.

## Provenance and peer review

This article was not commissioned and was peer reviewed.

## Ethics declaration

Written informed consent to take part in the study and to publish the article has been obtained from all participants or their legal representatives. The privacy rights of participants have been observed.

This study included organ or tissue donors.

This study includes human biological material and consent was obtained by donors, or their next of kin or legal representatives, for use in this study and for publication of the article. The samples used in this research were not sourced from executed prisoners or prisoners of conscience.

This study was performed in compliance with relevant laws, regulatory frameworks and guidelines where the research took place.

Ethics committee approval was not required under relevant laws and institutional guidelines. Ethics committee approval was not required for this case report in accordance with the relevant institutional guidelines. Written informed consent for publication was obtained from the patient.

This research follows the CARE guidelines and the CARE checklist.

## Declaration of generative AI and AI-assisted technologies in the writing process

During the preparation of this work, the authors used ChatGPT (OpenAI) solely to improve language and readability. After using this tool, the authors reviewed and edited the output as needed and take full responsibility for the content of the publication.

## Funding

This work did not receive any specific grant from funding agencies in the public, commercial, or not-for-profit sectors.

## Declaration of competing interest

The authors declare that they have no competing interest.
